# Artificial Intelligence Advances in the World of Cardiovascular Imaging

**DOI:** 10.3390/healthcare10010154

**Published:** 2022-01-14

**Authors:** Bhakti Patel, Amgad N. Makaryus

**Affiliations:** 1 Donald and Barbara Zucker School of Medicine at Hofstra/Northwell, Hofstra University, Hempstead, NY 11549, USA; bpatel10@pride.hofstra.edu; 2 Department of Cardiology, Nassau University Medical Center, East Meadow, NY 11554, USA; 3 Department of Cardiology, Northwell Health, Manhasset, NY 11030, USA

**Keywords:** artificial intelligence, information technology, cardiology, radiology, imaging

## Abstract

The tremendous advances in digital information and communication technology have entered everything from our daily lives to the most intricate aspects of medical and surgical care. These advances are seen in electronic and mobile health and allow many new applications to further improve and make the diagnoses of patient diseases and conditions more precise. In the area of digital radiology with respect to diagnostics, the use of advanced imaging tools and techniques is now at the center of evaluation and treatment. Digital acquisition and analysis are central to diagnostic capabilities, especially in the field of cardiovascular imaging. Furthermore, the introduction of artificial intelligence (AI) into the world of digital cardiovascular imaging greatly broadens the capabilities of the field both with respect to advancement as well as with respect to complete and accurate diagnosis of cardiovascular conditions. The application of AI in recognition, diagnostics, protocol automation, and quality control for the analysis of cardiovascular imaging modalities such as echocardiography, nuclear cardiac imaging, cardiovascular computed tomography, cardiovascular magnetic resonance imaging, and other imaging, is a major advance that is improving rapidly and continuously. We document the innovations in the field of cardiovascular imaging that have been brought about by the acceptance and implementation of AI in relation to healthcare professionals and patients in the cardiovascular field.

## 1. Introduction

In this age of technology, there have been numerous inventions created to expand the boundaries of medical treatment and diagnosis beyond their current capabilities. Among the technological advancements, artificial intelligence (AI) serves as a means to improve various technologies already in practice. Specifically, within the medical field, AI provides greater accuracy to help guide a patient’s course of treatment. Physicians are able to make clear initial decisions on how to treat patients presenting with specific symptoms. There is also a reduction in human error seen with the greater precision and automaticity capabilities of artificial intelligence. AI is beneficial for patients themselves as well by guiding patients to understand their symptoms through phone applications that detail whether patients need to go to the emergency room or their local doctor’s office based on acuity. Furthermore, AI has been used to strengthen the cardiac imaging modalities such as echocardiography, nuclear cardiac imaging, cardiovascular computed tomography, and cardiovascular magnetic resonance imaging. While AI has shown promise, limitations of AI include a lack of standardization and reproducibility of results as well as decision making and selection bias.

**Figure 1:**
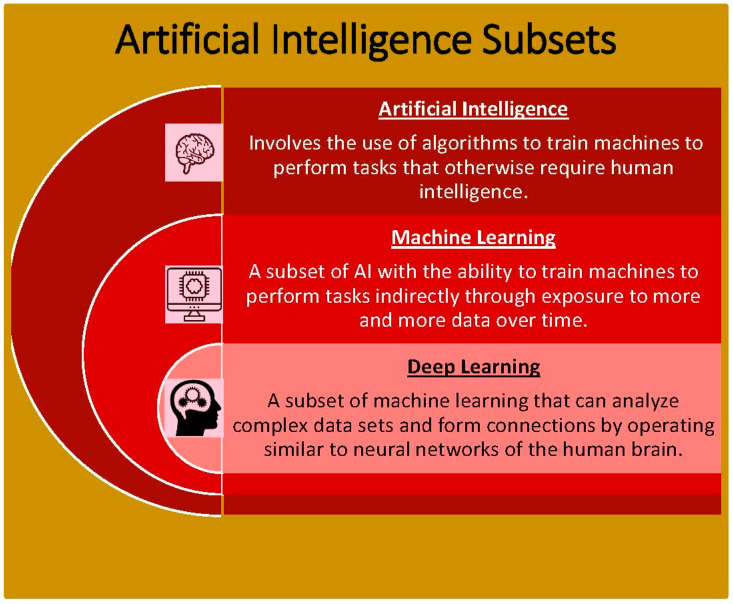
Artificial intelligence subsets into which the principles of AI can be divided.

Artificial intelligence refers to the all-encompassing ability of mathematical algorithms to train machines to mimic human intelligence. With the use of programmed algorithms, machines are able to complete tasks, execute decisions, and recognize images [1]. Within AI, machine learning is a subset (Figure 1) that identifies patterns among big datasets. It has the unique ability to automatically improve analysis over time with more usage of data and experience. Essentially, machine learning works by implementing algorithms to create a model from a sample dataset without directly programming the decisions needed to be made [2]. This is particularly useful in fields such as medicine where decisions are not predictable and vary in every individual patient. Machine learning provides the opportunity to handle complex data with the ability to become more accurate over time. Machine learning itself can be classified as supervised and unsupervised (Table 1). These two techniques are applied in different situations. Particularly, supervised learning refers to when models are trained to analyze algorithms based on reference data that have already been entered. Thus, as it works from a reference dataset and applies the same pattern to a new dataset, supervised learning is very accurate [3,4]. Unsupervised learning refers to finding patterns in data on its own without any given reference. This is advantageous in finding hidden patterns that have not already been identified [3,5]. Despite its advantages, machine learning has its limitations, especially apparent when applied to the field of medicine. Specifically, machine learning can lead to bias when it comes to analyzing the dataset. This is due to the way the algorithms are organized which is to become better with more exposure to previous datasets. Therefore, this decreases the variety of data that machines have to make information other than what was previously represented [6].

Furthermore, deep learning is a subset of machine learning. Deep learning uses artificial neural networks to allow machines to train themselves in accomplishing tasks. In other words, it can discover complex relationships that cannot otherwise be analyzed simply by an equation. It works to inspect and analyze an unlimited number of inputs at the same time [7]. A deep convolutional neural network (DCNN) is a specific type of neural network that involves restricted connectivity. Specifically, DCNN is often used for classification tasks as well as detection and localization [8]. DCNNs operate by involving convolutional layers, where each layer combines information from neighboring inputs to have a larger field of view. This is beneficial to finding patterns like visual pieces within an image, such as shapes and lines [9].

Deep learning is beneficial over traditional machine learning as it requires less data for training and has more accuracy [10]. In particular, deep learning is most valuable with pattern recognition and image identification, particularly when working with large image datasets. Therefore, it is most effective for cardiovascular imaging, such as echocardiography, angiography, and cardiac magnetic resonance. This is especially true as deep learning has the ability to parse through insignificant or noisy data [1]. Although deep learning is useful with image recognition, it is limited insofar as its algorithm cannot be efficiently applied for all types of datasets. For example, simpler machine learning would be easier to use for datasets that are more defined and structured. 

## 2. AI: General Medical Applications

The field of artificial intelligence allows for advancements to take place that expand the abilities of current technology. The goal of artificial intelligence is to create intelligence that has the ability for computers to solve problems and perform tasks, thus replicating the human mind [11]. AI involves the development of algorithms that can mimic the reasoning skills of humans in solving problems and deducing information in a methodical fashion. Over the years, AI has been applied to many different fields and used for a variety of purposes. Within medicine, AI has been used to improve diagnostic and treatment methods as well as efficiency with healthcare management [12]. For instance, most medical records are a collection of disorganized information hard to rifle through. However, with the application of AI, the information collected can allow physicians to understand a patient’s complete medical information prior to making medical decisions in real time. Specifically, algorithms that allow for the ability to search for patients with significant family history or susceptibility of chronic diseases transform the usage of electronic medical records [13]. More efficiently organized electronic medical records ultimately serve as a tool for personalized medicine and early detection of diseases.

In addition to programming algorithms, AI has also been applied to physical objects, such as medical devices and robots. For example, robotic-assisted surgeries are more often utilized to operate on patients [14]. The quality of care is drastically improved with the use of robots in surgery as incisions are more minimally invasive. This allows for patients to experience less pain after the surgery and have a shorter recovery time. The robotic surgical tool also serves to dissect, cut, and suture in a more precise fashion. With the addition of AI, surgical robots can identify the movements and patterns of a surgeon performing an operation and convert these into actions for the robot to execute on its own [15]. Additionally, the use of robots eliminates human error from surgeons, such as with hand tremors or accidental cuts [16]. In fact, of the 17 million surgical procedures performed in the United States, it was found that there were 400,000 operations with adverse outcomes attributed to human error [17]. Furthermore, Rajih et al. found robotic surgery error on the da Vinci surgical system in 4.97% of 1228 cases evaluated between 2012 and 2015 [18]. In this case, the use of AI-led robots allows for benefits such as improving the quality of care and providing accuracy and stability to prevent more human error.

## 3. AI: Cardiology Imaging Applications 

**Figure 2:**
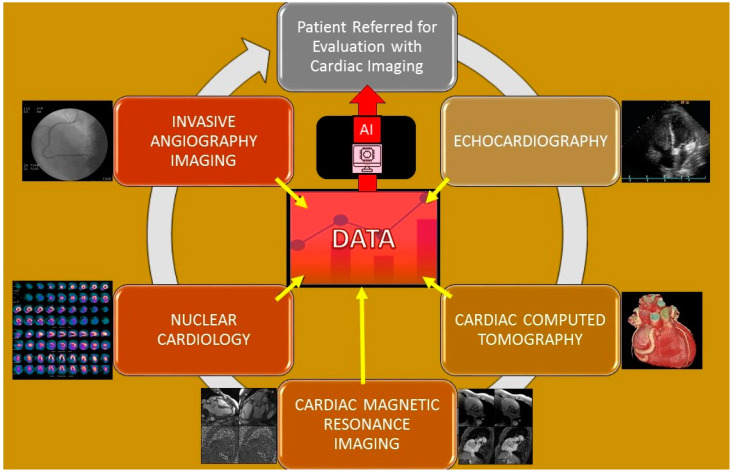
Cardiac imaging modalities that allow for the gathering of data that informs the formation of artificial intelligence that then is used for optimization of the evaluation of patients undergoing cardiac imaging.

Machine learning is a branch of AI that is particularly useful in the interpretation of cardiovascular imaging because it can combine and correlate information from different sources for a physician to interpret efficiently [19]. Specifically, machine learning has the ability to use a variety of different approaches to analyze a greater quantity of information. Coronary artery disease (CAD) is one of the most prevalent cardiovascular disorders and is responsible for one in every five deaths [20]. Coronary artery disease is generally diagnosed with radionuclide myocardial perfusion imaging (MPI). With the addition of machine learning to supplement the MPI results, the patient-specific risk stratification is improved. A study by Seetharam et al. found evidence that machine learning is greater than parametric statistical models in predicting the presence of obstructive CAD, the need for revascularization, and potential adverse risks [21]. Specifically, Arsajani et al. conducted a study that evaluated the MPI device’s accuracy of predicting CAD in 957 patients when used in adjunct with a learning algorithm compared to two experienced imaging readers. The results showed that the machine learning’s sensitivity and specificity was significantly superior compared to the experienced readers [19]. Multiple cardiac imaging applications and pertinent publications relating to them (Table 2 and Figure 2) are detailed below and lead to generation of data that inform artificial intelligence algorithms to allow for analysis and evaluation.

### 3.1. Echocardiography

Within the field of cardiology, AI has had a tremendous impact on how early and accurately patients are diagnosed as well as receive treatment. Echocardiography is a noninvasive diagnostic test that is performed on patients to detect or monitor the progression of cardiovascular diseases [17]. It is advantageous in visualizing the structure, function, and hemodynamics of the heart as well as any characteristic abnormalities. Specifically, echocardiography is beneficial as a cost-effective tool and can be performed at bedside rapidly with no known side effects [17]. On the other hand, a limitation of echocardiography is that it relies on a subjective interpretation of the images by the physician. Therefore, although obtaining the images is feasible with echocardiography, there is still a likelihood of an inaccurate diagnosis [23]. To address this limitation, AI provides the ability to produce accurate, consistent, and automated interpretations of echocardiograms [23]. Consequently, this reduces the likelihood of human error and allows physicians to come up with a precise treatment plan. The algorithms of AI also have the ability to accurately identify a wide variety of pathologies such as valvopathies and ischemia with coronary artery disease. In fact, Zhang et al. was able to use AI to accurately identify 96% of parasternal long axis imaging views from echocardiography [22]. The use of AI to improve the diagnostic ability of echocardiograms is still in its early stages and research is still in progress before it becomes more widespread [23]. 

It is often difficult to distinguish between several conditions on echocardiography. Narula et al. states how machine learning can be applied to echocardiography to help differentiate between hypertrophic cardiomyopathy and athlete’s heart [24]. Machine learning algorithms are also particularly beneficial to help streamline workflow and prevent errors from physicians reading images after experiencing fatigue and exhaustion. Specifically, Madani et al. applied a CNN algorithm model to 267 echocardiogram images with 15 standard views and trained the algorithm by using labeled images. The results found that the model was immediately able to identify the echocardiogram view with an accuracy of 97.8% as compared to 70.2–84% accuracy with readings by expert echocardiographers [25].

### 3.2. Cardiac Computed Tomography

In addition to echocardiography, computed tomography (CT) is also a valuable imaging tool for cardiovascular diseases. The CT scan produces images of the heart in various planes and allows for 3D image generation. It is particularly applicable for patients with suspected CAD as CT imaging allows physicians to noninvasively assess for calcium and plaque presence in the coronary arteries. This would indicate the presence of a blockage or narrowing in the arteries due to plaque buildup [26]. The amount of calcium in the vessels, also referred to as calcium score, can indicate the extent and prognosis of CAD. A study by Al’Aref et al. used machine learning algorithms to combine the calcium score and clinical factors to predict CAD in 35,281 patients. It was found that machine learning in conjunction with the coronary artery calcium score resulted in the most significantly accurate assessment of obstructive CAD from CT imaging compared to machine learning or calcium score alone [27]. Machine learning has been used to identify a variety of different pathologies on CT with accuracy [28].

Furthermore, machine learning allows for low-dose CT scans to be safer. Low-dose CT imaging brings concerns of increased exposure to radiation for patients that could not be solved by simply decreasing radiation levels as this would decrease the image quality [29]. As a result, to solve this issue, a machine learning framework was developed that allowed for reconstructing image parameters and denoising the quality of the image when low radiation was used. This resulted in improved image quality to equate to the regular-dose CT image quality, thus allowing patients to be exposed to less radiation while still obtaining a diagnostic result [30].

### 3.3. Cardiac Magnetic Resonance Imaging

Cardiac magnetic resonance imaging (MRI) is another noninvasive diagnostic tool for assessing cardiovascular diseases. Specifically, the MRI is considered the gold standard for assessing the ejection fraction and left ventricular volume [31]. In a study by Ruijsink et al., researchers found a high correlation between the deep learning algorithm and manual analysis of the left and right ventricular volumes, filling, and ejection rates [31]. In turn, automated measurements through deep learning were seen to be in strong agreement with the manual interpretation. Particularly, deep learning can be used to reconstruct cardiac images with better 3D visualization to identify disease patterns in association with the right ventricle. This is because the right ventricle is often not easily visualized in 2D with echocardiography. Laser et al. found that reconstruction of the right ventricle with echocardiography and cardiac MRI had incredible accuracy and reproducibility compared to the gold standard direct cardiac MRI [32]. Deep learning also allows for the extraction of specific features to be automated easily, such as identification of the right ventricle and pulmonary artery hypertension [19,33,34]. A study by Zhang et al. created a deep learning model that created the ability to obtain motion features from the left ventricle and discriminate between ischemic regions on a nonenhanced cardiac MRI. This deep learning framework is beneficial as it allows for confirmation of chronic myocardial infarctions on MRI [35].

Cardiac MRI has increasingly been used as a noninvasive imaging tool over the years. It results in acquiring cross-sectional images aligned with the heart axes. This can pose as an additional challenge when reading MRI results as medical imaging experts require detailed knowledge on cardiac anatomy. Conventionally, the heart is automatically localized to the center of the image, which does not account for the diversity in various patients’ anatomy. This assumption leads to lower sensitivity and potential errors in imaging results [9]. Kabani et al. introduced CNN, or deep neural network application, which allows for localizing and detecting a region of interest on an MRI interest. Normally, most localization networks have a bounding box around the region of interest. In this case, the CNN neural network applied a classification task where each pixel in the image was a separate class. Then, the CNN was trained to determine where the object was in the image and classify the pixels as in the background or in the bounding box. Specifically, this neural network considered the problem as a classification task where the pixels were classified as in the background or in a box [8].

### 3.4. Nuclear Cardiology

Nuclear cardiology uses noninvasive techniques to measure blood flow through the heart. This test is particularly applicable when diagnosing coronary artery disease and possible ischemia, or lack of oxygen to the heart due to decreased blood flow. There are two types of nuclear cardiology tests that can be performed, the cardiac SPECT and PET-CT. In both tests, a PET scan is formed following injection of radioactive chemicals into the bloodstream via IV [36]. Artificial intelligence, particularly deep learning, can also be applied to nuclear cardiology in order to address disparities regarding the diagnostic ability of SPECT [19]. Deep learning allows for a greater ability to analyze images by identifying high dimensional patterns. Juarez-Orozco et al. applied deep learning to evaluate perfusion polar maps in ischemia by PET. It was found that deep learning had an area under the receiver-operating curve (AUC) of 0.90, which was better than all comparator models [19]. Additionally, Hu et al. implemented the subset of AI, machine learning, to predict the likelihood of early coronary revascularization within 90 days after SPECT imaging. When comparing the AUC of machine learning with the standard quantitative analysis, it was found that the AUC of early coronary revascularization prediction was higher than and outperformed that of standard quantitative analysis [37].

### 3.5. Angiography Imaging

Another imaging tool used in the field of cardiology is invasive angiography imaging. This is considered the reference standard when diagnosing obstructive coronary artery disease as it provides a detailed outlook on the structure and function of the heart’s blood vessels. Despite its benefits, there are risks associated with the invasive angiography procedure including serious complications as well as expensive costs, exposure to high radiation, and discomfort [38]. As such, Wolterink et al. validated a feasible method to obtain reduced radiation dose CT images by training a deep learning model [39]. This shows how deep learning can help improve diagnostic imaging tools as well to make procedures safer for patients while also providing more accurate results. 

Furthermore, cardiac computed tomography angiography (CCTA) is another method to diagnose coronary artery disease. Although CCTA can be used to rule out CAD, there are many drawbacks to using this diagnostic tool as it overestimates the amount of stenosis of the vessel and takes a lot of time to yield results. However, with the addition of AI, CCTA can be significantly improved to result in a more accurate evaluation of coronary stenosis, plaque characterization, and degree of myocardial ischemia [40]. For example, van Hamersvelt et al. evaluated the addition of deep learning algorithm to analyze the left ventricular myocardium in CCTA for degree of stenosis. It was found that there was improved diagnosis and identification of patients with functionally significant coronary artery stenosis when using CCTA in combination with deep learning analysis. Specifically, sensitivity and specificity of results were 84.6% and 48.4%, respectively [41].

Motwani et al. applied machine learning to evaluate 5-year all-cause mortality in patients undergoing CCTA [42]. Specifically, 10,030 patients with possible CAD underwent CCTA as part of their standard of care. Machine learning was then applied to predict 5-year mortality of these patients using the CCTA data. After comparison of a 5-year follow-up from these patients via the CCTA international multicenter registry, it was found that ML combined with CCTA data was significantly better at predicting patient prognosis for the next 5 years compared to CCTA metrics alone [42].

ML-based fractional flow reserve-computed tomography (FFR-CT) is increasingly used in diagnosing CAD. Specifically, FFR-CT is a noninvasive procedure that generates a 3D image of the patient’s coronary arteries [43]. An FFR measurement refers to identifying the ratio between the maximum blood flow possible in a diseased coronary artery and maximum flow in a normal coronary artery. An FFR of 1.0 is considered normal whereas an FFR of less than 0.75–0.80 is associated with myocardial ischemia [44]. A study by Jiang et al. evaluated the features and severity of coronary calcification by ML-based CCTA-derived FFR, or FFR-CT. In this study, 442 patients went through CCTA, ML-based FFR-CT, and invasive FFR and the results were compared. It was found that ML-based FFR-CT had an accuracy of 0.90 in determining calcification lesions as compared to invasive FFR. Additionally, CT-FFR generally had higher accuracy in diagnosis and differentiating ischemia in blood vessels as compared to CCTA by itself [45].

A study by Yang et al. analyzed the relation of stenosis and plaque characteristics with myocardial implications. The study analyzed 1013 vessels via fractional flow reserve measurement and CT angiography. Then, Yang et al. incorporated machine learning to identify the features associated with a low FFR and the patient prognosis. In this case, machine learning was beneficial in categorizing characteristics of blood vessels with a low FFR. The six functionally relevant features found included minimum lumen area, percent atheroma volume, fibrofatty and necrotic core volume, plaque volume, proximal left anterior descending coronary artery lesion, and remodeling index [46].

### 3.6. Intravascular Imaging

Intravascular imaging is performed by using a specialized catheter-based intravascular ultrasound (IVUS) or optical coherence tomography (OCT) that allows for providing a real-time visual of the inside of a coronary artery. Particularly, it shows the degree of narrowing or thickening of an artery and a visual of the lumen of the artery. Intravascular ultrasound is often used to gain a better insight into the nature of the plaque in the artery as well as in the placement of stents [47]. This imaging technique is invasive and involves great expertise to place the catheters inside the coronary arteries. Researchers have incorporated artificial intelligence to increase the speed of diagnosis and interpretation of intravascular imaging in real time [48]. Specifically, the artificial intelligence algorithm is exposed to multiple images and given information on each image, such as the vessel’s geometry and distribution of different tissue types. Therefore, as it is exposed to more images, the AI algorithm can easily and quickly interpret the image created by the intravascular imaging and discern a diagnosis. 

IVUS imaging creates an image with low resolution but with high tissue penetration while OCT imaging creates an image with higher resolution but limited tissue penetration. As these two intravascular imaging modalities have their differences, artificial intelligence can connect the two results together. Specifically, AI processes data from IVUS and OCT images into a single imaging procedure to allow physicians to review all the data at once [48]. This is beneficial as it allows for a more rapid, comprehensive evaluation of any damaged arteries.

### 3.7. Software Programs in Clinical Practice That Employ AI

Artificial intelligence is already being used in clinical practice by physicians today. There are software programs, such as IBM Watson^®^, that help organizations automate complex processes to improve efficiency and effectivity. IBM Watson^®^ includes Merge Healthcare^®^, which provides medical imaging artificial intelligence solutions to help physicians with patient care. Specifically, Merge PACS™ is an artificial-intelligence-ready workflow platform that eases the physician workload of reading and understanding numerous dense images [49]. This is extremely beneficial as physicians have received an increasing number of images to read over the years, including as many as 100,000 images a day [50]. Therefore, artificial intelligence and computer programming offered through Merge Healthcare^®^ serves to provide a more rapid and automated diagnosis for patients.

## 4. Limitations of Artificial Intelligence

Overall, AI applications mimic human intelligence with the purpose of solving problems or making decisions. AI has many advantages with its accuracy, cost-effectiveness, and reliability. However, there are still some limitations to AI, especially with its application in the medical field. Specifically, the gold standard for clinical reasoning in decision making should still be at the physicians’ discretion. Since AI results in producing automated decisions, this can lead to a decision-making bias as physicians can be more likely to trust diagnostic test results by AI-led machines without intense scrutiny [33]. Consequently, there is a gray area as to with whom the responsibility lies in the case of an error. Data interpretation with AI can be susceptible to selection bias as well [34]. This is because the results AI produces are dependent upon the data entered. Therefore, if there is poor data entry, the results lead to invalid assumptions without a fair, accurate representation. Furthermore, another limitation of newer models of AI is the ability to reproduce and standardize the method [34,51,52]. It is difficult to compare diagnostic results from different providers if they are analyzing the data with varying techniques.

## 5. Future Applications of Artificial Intelligence

As newer techniques are emerging, AI is constantly expanding beyond its limits and capabilities. Within the field of medicine, it has the potential to lead to newer advances in drug therapies as well as diagnoses of diseases at an earlier stage [34]. It is anticipated that AI will have the ability in the future to fully automate reading echocardiography images and detecting pathology [32]. Furthermore, it allows treatment plans to become more standardized based on an automated process. With AI completing tasks at a quicker rate, physicians have more time to be free from mundane tasks such as data input and electronic health records to focus on educating the patient and fostering a stronger patient–physician relationship [32]. Although AI holds great potential for the future of medicine, physicians should still be responsible for making the final clinical judgment.

## 6. Conclusions

Artificial intelligence allows for the potential to expand and improve medical technologies for better patient care. Specifically, the ability of the algorithms to make diagnoses more accurate is useful for physicians to detect diseases earlier in their course to plan for the right treatment action. Within AI, the branch of machine learning has been prevalent in the field of cardiology. This is because there are a variety of imaging tools implemented when conducting a patient workup. In the future, AI will continue to expand and become more accurate in giving an ideal diagnosis for improved decision making as technology progresses and the dataset available to form algorithms and identify patterns becomes larger.

## Figures and Tables

**Table 1:** Methodology of supervised and unsupervised learning within machine learning.

Machine Learning Classification	Types of Problems Each Classification Is Used for
**Supervised Learning**—Uses reference data to analyze algorithms and apply the algorithms to a similar dataset [3]	**Classification**—Utilizes an algorithm to assign a dataset into specific categories. Specifically, draws conclusions on how specific categories in the dataset should be labeled. [4]
	**Regression**—Analyzes the relationship between dependent and independent variables, particularly for making projections [4]
**Unsupervised Learning**—Identifies hidden patterns in data without any given reference [3]	**Clustering**—Organizes unlabeled data based on similarities and differences [5]
	**Dimension Reduction**—Reduces the number of data inputs while preserving the data integrity; applied when there is an increased number of features or dimensions in a dataset [5]

**Table 2:** Pertinent publications regarding artificial intelligence.

Pertinent Publications Related to Artificial Intelligence in the Field of Cardiovascular Imaging	Findings in Publication
Improved accuracy of myocardial perfusion single-photon emission computed tomography [SPECT] for the detection of coronary artery disease using a support vector machine algorithm	Arsajani et al. found that the accuracy of predicting CAD with an MPI device improved significantly when in adjunct with a learning algorithm [22]
Fully Automated Echocardiogram Interpretation in Clinical Practice	Zhang et al. determined 96% accuracy in identifying images with echocardiography [22]
Machine learning of clinical variables and coronary artery calcium scoring for the prediction of obstructive coronary artery disease on coronary computed tomography angiography: analysis from the CONFIRM registry	Al’Aref et al.’s results showed a significantly more accurate assessment of obstructive CAD from CT imaging using machine learning with the coronary artery calcium score [21]
Cardiac Imaging on the Cusp of an Artificial Intelligence Revolution	Laser et al. determined that the right ventricle reconstruction with echocardiography and cardiac MRI had more accuracy compared to the gold standard direct cardiac MRI [23]
